# Russian collection of *Brucella abortus* vaccine strains: annotation, implementation and genomic analysis

**DOI:** 10.3389/fvets.2023.1154520

**Published:** 2023-06-21

**Authors:** Olga Prasolova, Ekaterina Krylova, Alexandra Bogomazova, Irina Soltynskaya, Oleg Sklyarov, Veronika Gordeeva, Irina Timofeeva, Anton Motorygin, Alexander Panin

**Affiliations:** ^1^The Russian State Center for Animal Feed and Drug Standardization and Quality (VGNKI), Moscow, Russia; ^2^The Lopukhin Federal Research and Clinical Center of Physical-Chemical Medicine of FMBA of Russia, Moscow, Russia

**Keywords:** vaccines, brucellosis, strains, selection, NGS, bioinformatics analysis annotation

## Abstract

Over the past 10 years, immunization of cattle in Russia has been performed using vaccines from *Brucella abortus* strains 82, 19 and 75/79. To prevent brucellosis in small ruminants, two vaccines have been used, from the *Brucella melitensis* strain REV-1 and the *B. abortus* strain 19; note that twice as many animals have been immunized with the former vaccine than with the latter vaccine. The disadvantage of using these preparations is the formation of prolonged post-vaccination seropositivity, which is especially pronounced in animals after immunization with vaccines from *B. abortus* strain 19 and *B. melitensis* strain REV-1. This study aims to perform the whole genome sequencing of *Brucella* vaccine strains from the Russian collection. A bioinformatics analysis of the genomic data proved that the vaccine strains 75/79AB, 82, R-1096, and the KV 17/100 belong to ST-2, 104 M to ST-1, KV 13/100 to ST-5. This analysis allowed us to characterize vaccine strains’s phylogenetic relationships and to prove the close relation of vaccine strains 75/79AB, 82, R-1096. Also, we defined candidate mutations in genes *pmm, wbdA, wbkA, wboA,* and *eryB*, which could be responsible for the attenuated virulence of vaccine strains. The complete genomic sequences of *B. abortus* strains make further studies of bacterial pathogenicity determinants and virulence phenotype feasible, as well as their use in quality control of animal medicines.

## Introduction

The development of measures for specific prophylaxis of brucellosis in the world began almost immediately from the moment of discovery of its causative agent. It was started by Bang who for the first time isolated microorganisms from the amniotic fluid of a cow that suffered abortion, selected the correct medium and determined optimal conditions for their cultivation (1). By infecting animals, he also proved that the isolated microorganisms are the causative agent of infectious abortion; he later noted that infected cows acquired certain immunity to this disease. It was further evidenced by observing cases of self-recovery of animals from infectious abortion. Based on this, Bang concluded that it is possible and necessary to develop measures for specific disease prophylaxis. Many researchers began to work in this direction, and, first of all, Bang himself.

In 1906, Bang reported the results of experiments which allowed him to form an immunity in pregnant sheep, goats and cows by inoculating them with live virulent cultures of *Brucella*. In 1909, he for the first time in the field experiments performed the intravenous administration of live *Brucella* broth cultures to animals, as a result of which they developed a high degree of immune response, which, however, was accompanied by signs of anaphylactic shock.

Since 1910, in England, Germany, Denmark, United States, and Argentina, agar swabs of *Brucella* cultures were used for mass vaccination of young and non-pregnant cows. Although the number of abortions in animals was slightly decreasing, the damage from the infection was about the same as in the natural course of the disease. The widespread use of virulent cultures caused abortions in pregnant animals and essentially led to their massive artificial infection. Thus, vaccinated animals presented an epizootic and epidemic danger.

Due to this, later on strains with attenuated virulence were used for the production of vaccines. Attenuated virulence was achieved by treating virulent cultures of *Brucella* with chemical, physical and biological methods. Some of the researchers were able to achieve significant success.

In 1934, Corner et al. (2) worked with a culture of *Brucella abortus* strain *19* isolated by Buck in 1923 from the milk of a cow of the third calving and found that after storage for one year at room temperature the culture spontaneously reduced its virulence. The authors were able to select an immunogenic and stable strain which subsequently was named Buck-19 [B-19] in honor of the author of the original culture. From that moment on, a new stage in the development of anti-brucellosis vaccines has begun, while the vaccine from strain 19 remains the standard of immunogenicity until now.

*Brucella* species are characterized by high invasiveness which allows them to successfully multiply in macrophages and lymphocytes (3). At the same time, *Brucella* lack virulence factors such as the capsule, flagella, fimbriae, pili, plasmids, toxins, exotoxins, cytolysins, and secreted proteases found in other bacteria (4, 5). Various virulence factors, the mechanism of evasion from the host defense systems and the method of intracellular survival of *Brucella* were reviewed in detail in (6). It was shown that the virulence of *Brucella* can be determined by: lipopolysaccharide (LPS) (7), β-glucan (β-cyclic glucan) (8, 9), BvrS/BvrR, BacA (10), outer membrane proteins (Omps) (11), BmaC (12), SagA (13), BtaE (14), BetB (15), MucR and T4SS genes found in *vir*B operon (16). The vital virulence factor, LPS, determines the morphology of *Brucella* colonies in culture. The smooth phenotype (S-form) is formed due to complete LPS, consisting of lipid A, core oligosaccharide, and O-side chains of the polysaccharide. The strains of *Brucella* with attenuated virulence often form rough colonies (R-form) due to a deficiency of O-side chains of the polysaccharide (17).

In accordance with the List of Prokaryotic Names with Standing in Nomenclature (LPSN), the causative agent of brucellosis is assigned to the genus *Brucella*, family *Brucellaceae*, order *Rhisobailes*, class *Alphaproteobacteria*. The genus *Brucella* consists of 13 independent species. A total of six typical and seven new *Brucella* species were identified in a wide range of susceptible hosts. There are seven species that infect terrestrial animals, including *B. abortus, Brucella melitensis, Brucella suis, Brucella ovis, Brucella canis, Brucella neotomae,* and *Brucella microti* (18); two more species, *Brucella ceti* and *Brucella pinnipedialis*, infect marine mammals (19). *Brucella papionis* was isolated from baboons and *Brucella vulpis* from red foxes (20). Seven biovars have been recognized for *B. abortus*, three for *B. melitensis* and five for *B. suis*. Other species are not subdivided into biovars. *Brucella* nomenclature is based on the species of the main hosts (21). As the list of species grows, it is important to identify more effective prophylaxis measures to control the spread of the disease in humans.

Considering the complexity of differentiation of various species of *Brucella* bacteria, including using 16sRNA analysis for typing and comparison with other strains, we carried out whole genome sequencing and phylogenetic analysis by single nucleotide polymorphisms (SNPs) in order to comparatively assess the molecular-genetic characteristics of strains and analyze alternative options for the production of vaccines in Russia.

## Materials and methods

### Object of study

The strains for this study were obtained from the All-Russian State Collection of Microorganisms Used in Veterinary Medicine and Animal Husbandry of VGNKI: *B. abortus* 82, *B. abortus* 75/79-AB, *B. abortus* 104 M, *B. abortus* KV 17/100, *B. abortus* R-1096, *B. abortus* KV 13/100. To construct a phylogenetic tree based on SNP loci, we selected genomes of vaccine strains and field isolates from the NCBI database that were assembled completely or at the scaffold level (Table 1).

**Table 1:** List of *Brucella abortus* strains used for phylogenetic analysis.

Strain	MLST	Assembly	Level	Geographic location	Host	Reference
104М^*^	ST-1	GCA_001296965.1	Complete	China	Cattle, vaccine strain	Yu et al. (22)
9-941	ST-1	GCA_000008145.1	Complete	USA	Cattle	Halling et al. (23)
BDW	ST-1	GCA_000740135.1	Complete	USA	n.d.	Minogue et al. (24)
15500	ST-2	GCA_002291225.1	Chromosome	Italy	*Bubalus bubalis*	Paradiso et al. (25)
C68	ST-2	GCA_000740195.1	Complete	USA	n.d.	Minogue et al. (24)
63 75	ST-2	GCA_000740295.1	Complete	USA	n.d.	Minogue et al. (24)
82	ST-2	GCA_000473805.1	Scaffold	Russia	Cattle, vaccine strain	Shevtsov et al. (26)
I-182	ST-2	GCA_016484075.1	Contig	Russia	Cattle	n.d.
BC95	ST-2	GCA_000477635.1	Scaffold	Spain	Cattle	n.d.
67/93	ST-2	GCA_000370165.1	Scaffold	Iraq	*Bubalus bubalis*	n.d.
03-4923-239-D	ST-3	GCA_000480075.1	Scaffold	n.d.	n.d.	n.d.
NCTC 10505	ST-4	GCA_000740175.1	Complete	USA	n.d.	Minogue et al. (24)
870	ST-4	GCA_000740215.1	Complete	USA	n.d.	Minogue et al. (24)
RB51	ST-5	GCA_011801185.1	Complete	USA	Vaccine strain	Bricker et al. (27)
A13334	ST-5	GCA_000238175.1	Complete	South Korea	Cattle	Kim et al. (28)
S19	ST-5	GCA_000018725.1	Complete	USA	Vaccine strain	Crasta et al. (29)
2,308	ST-5	GCA_000054005.1	Scaffold	USA	Standard laboratory strain	Chain et al. (30)
BFY	ST-6	GCA_000740315.1	Complete	USA	n.d.	Minogue et al. (24)
BER	ST-6	GCA_000740155.1	Complete	USA	n.d.	Minogue et al. (24)
F1/06-B21	ST-6	GCA_000370365.1	Scaffold	Zimbabwe	Cattle	n.d.
F10/05–11	ST-28	GCA_000370385.1	Scaffold	Portugal	cattle	n.d.
84/26	ST-29	GCA_000370265.1	Scaffold	Mexico	*Homo sapiens*	n.d.
85/69	ST-31	GCA_000370285.1	Scaffold	India	Cattle	n.d.
80/28	ST-32	GCA_000370245.1	Scaffold	Chad	Cattle	n.d.
78/14	ST-34	GCA_000370185.1	Scaffold	Chad	Cattle	n.d.
78/32	ST-36	GCA_000370205.1	Scaffold	Senegal	Cattle	n.d.
80/101	ST-36	GCA_000370225.1	Scaffold	Nigeria	n.d.	n.d.
88/217	ST-37	GCA_000370345.1	Scaffold	Mozambique	Cattle	n.d.
63/294	ST-38	GCA_000370065.1	Scaffold	Kenya	n.d.	n.d.

^*^104 M is a vaccine strain applied for brucellosis prevention for the last 60 years in China. This strain was first isolated in former Soviet Russia (Li et al., 2015). n.d.–no data.

### Microbiological cultivation methods

Bacteria were cultivated on *Brucella* agar at 37°C for 48 h. A microbial suspension with a concentration of 1.7 × 10^9^ CFU/mL was disinfecte—d by adding sodium merthiolate (0.01%) and incubating at 56°C for 30 min. Additional incubation with CO_2_ was not performed.

### Method for studying immunogenicity

The immunogenicity of the microorganism strains was studied in guinea pigs by introducing 10–15 minimal infectious doses of the culture of the control virulent strain (*B. abortus* 54 VGNKI).

### Extraction of genomic DNA

Total genomic DNA was isolated using the DNA-sorb-V Kit (Central Research Institute for Epidemiology, Russia) in accordance with the manufacturer’s instructions. DNA concentration was measured on a Quantus fluorimeter (Promega, United States) using the QuantiFluor^®^ONE dsDNA System Kit (Promega, United States).

### Genome sequencing, bioinformatics analysis

DNA libraries were prepared using the Nextera XT DNA Sample Preparation Kit in accordance with the manufacturer’s instructions. Whole genome sequencing was performed on a MiSeq system (Illumina, United States) in accordance with the standard operating procedure.

For bioinformatics analysis of data of whole genome sequencing and *de novo* genome assembly, the following programs were used: FastQC 0.11.17 (31), Trimmomatic v.0.36 (32), SPAdes 2.11.1 (33), QUAST 4.6.3 (34), MAUVE v.20150226 (35). Bacterial species identification and multilocus sequence typing (MLST) were performed *via* the online service of the Center for Genomic Epidemiology of the Danish University of Technology (CGE) using the KmerFinder server (version 3.0.2) and MLST server (version 2.0.4) (36, 37). Annotation of bacterial genomes was performed using the RAST server (38).

The search for antibiotic resistance genes was carried out using the ResFinder 4.1 online service (39) as well as the Arg-ANNOT, CARD and NCBI BARRGD databases (40). Identification was carried out with the ABRicate program (41) using BLASTN and BLASTX against nucleotide and amino acid sequences from various databases. To search for the main virulence factors in bacterial genomes, the Virulence Factor Database (VFDB) (42) and Victors database were used (43); to search for integrons, the IntegronFinder v5 program was used (44). For phylogenetic analysis, the kSNP v.3.1.2 program was used (45). For visualization, the service iTOL v.6.4.3 (Interactive Tree of Life) was used (46).

## Results and discussion:

An analysis of epizootic data from 1946 to the present day shows that brucellosis in cattle on the territory of Russia was not regx+istered only in the Kamchatka. Circulation of a total of 6 species of *Brucell*a was detected: *B. abortus, B. melitensis, B. suis, B. neotomae, B. ovis* and *B. canis*. The most virulent for humans are *B. melitensis, B. abortus* and *B. suis*, and to a lesser extent *B. canis, B. ceti* and *B. pinnipedialis*. Therefore, when conducting laboratory diagnostics for brucellosis, it is important to perform not only the isolation of the culture of the pathogen, but also the identification of the specific species. To use as part of the composition for specific prophylaxis in animals, different authors proposed 25 strains of microorganisms of the genus *Brucella*, mainly *B. abortus, B. melitensis* and rarely *B. suis*. So far, however, only 9 of them were used in the composition of vaccines that were used to treat farm animals (Table 2).

**Table 2:** The vaccine strains of *Brucella abortus* from the Russian collection.

Strain	Put into practice	Reference
*В. abortus* 19	+	Buck (47)
*B. melitensis* Rev-1	+	Elberg et al. (48)
*B. abortus RB-51*	+	Schurig et al. (49)
*В. abortus* 104 М^*^	+	Shumilov et al. (50)
*В. abortus* 82^*^	+	Salmakov (51)
*B. abortus* 75/79-АВ^*^	+	Nikiforov et al. (52)
*B. abortus* KV 17/100^*^	− (mini-batch)	Kalmykov et al. (53)
*B. abortus* KV 13/100^*^	+	Kalmykov (54)
В. abortus R-1096^*^	+	Salmakov et al. (55)

^*^The whole genome of the strain was sequenced in this study.

For the mass prophylaxis of brucellosis in cattle, live vaccines from *B. abortus* strains 19, 82 and 75/79-AB were used in different volumes, in small ruminants—from *B. melitensis* strain Rev-1 and *B. abortus* strain 19. It is important to note that there exist no *Brucella* vaccines for medical and veterinary use guaranteed to protect all those immunized. The degree of their protection depends on the virulence and dose of field *Brucella* cultures (56). Until 1952, the measures against brucellosis in the Russia consisted of conducting diagnostic studies and removing sick animals from herds, without the use of anti-brucellosis vaccines. Due to the aggravation of the epizootic situation in 1953, the system of anti-brucellosis measures was expanded: it included immunization of animals with a vaccine from the *B. abortus* strain 19. This vaccine was used until 1975. During the period of active implementation of the vaccine, the disease was eliminated from many farms and even entire regions. However, in regions with a wide spread of brucellosis, the effectiveness of these sanation measures was insufficient. This was primarily due to the high agglutinogenicity of the vaccine. Agglutinins and complement-binding antibodies are preserved in the bodies of repeatedly immunized animals for up to 5–8 years, which makes it extremely difficult to differentiate such animals from those with brucellosis.

**Figure 1:**
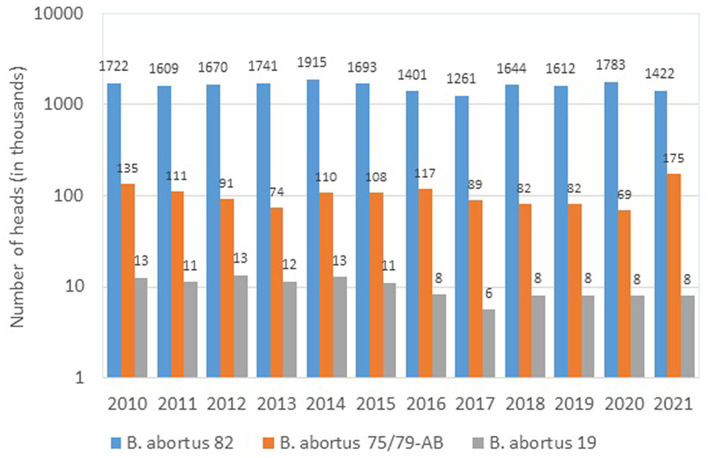
*Brucella* strains in the composition of vaccines used for the prophylaxis of brucellosis in cattle in Russia (2010–2021, log scale).

**Figure 2:**
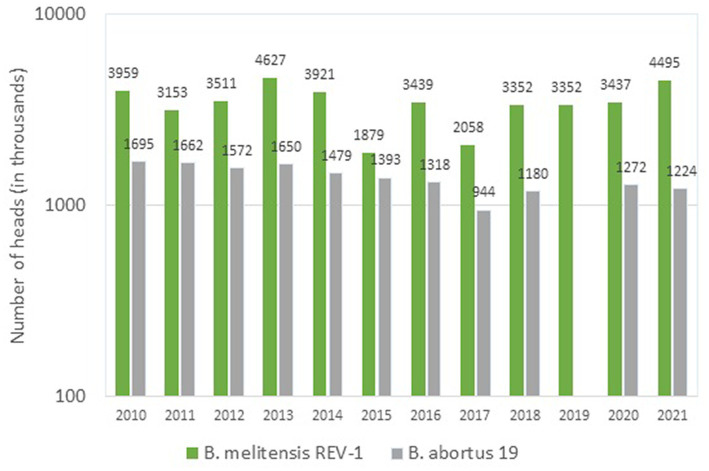
*Brucella* strains in the composition of vaccines used for the prophylaxis of brucellosis in small ruminants in Russia (2010–2021, log scale).

According to official data (57), in the period of 2010–2021 three vaccines were used in Russia for immunization of cattle (Figure. 1), the main of which was the vaccine from the *B. abortus* strain 82. To prevent brucellosis in small ruminants, two vaccines were used (Figure. 2); among them, the vaccine from the *B. melitensis* REV-strain 1 was used to immunize twice as many animals as the vaccine from the *B. abortus* strain 19.

As seen from the Figures 1, 2, over the past 11 years up to two million heads of cattle have been immunized annually with a vaccine from the *B. aborus* strain 82 (from 1,261.3 thousand heads in 2017 to 1,915.3 thousand heads in 2014). Despite that, today this vaccine, as almost any other live vaccine, does not fully meet the requirements of veterinary practice due to its abortogenic properties. Nonetheless, to a large extent, this problem can be solved by immunizing heifers at the age of 4–6 and/or 12–14 months. Subsequently, sensitized animals can be immunized against brucellosis during pregnancy, but only if the infection with field cultures of *Brucella* is completely ruled out. However, the bulk of the breeding stock of cattle is immunized after calving.

The *B. abortus* strain KV 17/100-VGNKI was obtained by targeted selection for cultural, morphological, biochemical and antigenic properties is very promising for the production of an inactivated vaccine with an adjuvant when constructing and testing a mini-batch (58). This vaccine does not induce the synthesis of S-*Brucella* antibodies in diagnostic titers in animals not infected with *Brucella* but induces it in cattle with a latent form of brucellosis, which allows, due to the rapid removal of such animals from herds, to accelerate the recovery of affected farms from brucellosis. At the same time, it does not have abortogenic properties and can be used for immunization of pregnant cows and heifers. Moreover, since it is in an inactivated state, it does not pose an environmental hazard.

Next, we performed whole genome sequencing of six vaccine strains: *Brucella abortus* strain 82, *B. abortus* strain 75/79-AB and *B. abortus* strain R-1096; *B. abortus* strain KV 17/100 and *B. abortus* strain 104 M used as an antigen to provoke latent forms of brucellosis; and *B. abortus* strain KV 13/100 used to control the immunogenic activity of inactivated vaccines (Table 3).

**Table 3:** Immunogenic properties of *Brucella* strains subjected to whole genome sequencing.

Strain	Purpose and state of strain in vaccine		Species of animal	Virulence	^a^Immunogenicity, %	^b^Dose, bil. m.c.	Type of colonies	Practical application
R-1096	Antigen for provoking of latent forms of brucellosis	Live	Cattle	Attenuated	40–60	100	R-form	−
KV 17/100	Adjuvant-vaccine	Inactivated	Cattle	Attenuated	50–70	450	R-form	+ (mini-batch)
KV 13/100	Control	Live	−	Attenuated	−	−	R-form	−
82	Vaccine	Live	Cattle	Attenuated	70–80	100	SR-form	+
75/79-АВ	Vaccine	Live	Cattle	Attenuated	70–80	100	SR-form	+
104М	Vaccine	Live	Cattle	Attenuated	90–100	80	S-form	−

a The percentage of immunogenicity is indicated according to the results of experiments on guinea pigs with the administration of 10–15 minimal infectious doses of a culture of the virulent strain *B. abortus* 54 VGNKI.

b bil. m.c., billion microbial cells.

### The bioinformatics analysis of genomic data of *Brucella abortus* strains

The genomes of all *Brucella* species have the same size and genome map (59). The average size of the genome which consists of two ring chromosomes is approximately 3.29 Mb.

The quality of sequencing data (FASTQ files) was assessed using the FastQC_0.11.17. The removal of technical sequences and low-quality nucleotides was performed in the Trimmomatic v.0.36 with the following ILLUMINACLIP parameters: NexteraPE-PE.fa: 2:30:10, SLIDINGWINDOW: 4:15, MINLEN: 50. The *de novo* assembly of bacterial genomes was performed using SPAdes 2.11.1 with the sequencing error correction and automatic selection of k-mer length (21, 33, 55, 77, 99). Contigs shorter than 500 bp were excluded from further analysis. The assembly with the smallest number of contigs and the largest N50 value was chosen as the best one. The main characteristics of the assembly were obtained using the QUAST 4.6.3 and are presented in Table 4.

**Table 4:** Main characteristics of draft genome assemblies of *B. abortus strains.*

Strain	Number of contigs	Max length of contig, bp	Total length of contigs, bp	N50	GC content, %
R-1096	17	1,101,404	3,247,278	530,773	57.3
75/79-АВ	22	714,196	3,261,112	443,659	57.2
82	21	883,913	3,260,553	462,459	57.2
KV 17/100	18	1,098,592	3,257,570	462,111	57.2
KV 13/100	23	518,914	3,263,681	316,877	57.2
104М	26	545,702	3,266,856	309,189	57.2

To identify a bacterial species using the assembled contigs, we used the method of searching for common k-mers. Multilocus typing of the strain was carried out for the *aroA, cobQ, dnaK, gap, glk, gyrB, int_hyp, omp25*, and *trpE* loci. The MLST profiles and loci sequences were obtained from the PubMLST database (23). Genotyping data are shown in Table 5.

**Table 5:** Results of genotyping of *B. abortus* strains.

Strain	MLST	KmerFinder
R-1096	ST-2	NZ_CP008774.1 *Brucella abortus* strain BAB8416
75/79-АВ	ST-2	NZ_CP008774.1 *Brucella abortus* strain BAB8416
82	ST-2	NZ_CP008774.1 *Brucella abortus* strain BAB8416
KV 17/100	ST-2^*^ (gyrB^*^)	NZ_CP007705.1 *Brucella abortus* bv. 9 str. C68
KV 13/100	ST-5	NZ_CP030751.1 *Brucella abortus* strain A19
104М	ST-1	NZ_CP009625.1 *Brucella abortus* 104 M

^*^The strain KV 17/100 corresponds to ST-2 at eight out of nine loci with 100% identity, and at the gyrB locus, the identity is 99.79% due to single SNP.

Annotation of genomes was performed using the RAST server on the SEED, an open platform for comparative analysis of genomes. The preliminary contigs were ordered using the MAUVE v.20150226 relative to the corresponding sequences indicated in Table 5 in the KmerFinder column.

To analyze contig sequences for the presence of various antibiotic resistance genes, the following criteria were used: >95% identity, >80% minimum intersection length. No antibiotic resistance genes were identified in any of the strain. Genome analysis did not reveal the presence of integrons in any of the strains.

### Phylogenetic analysis of *Brucella abortus* vaccine strains

For typing and comparison with other strains, a phylogenetic analysis was carried out using single nucleotide substitutions.

**Figure 3:**
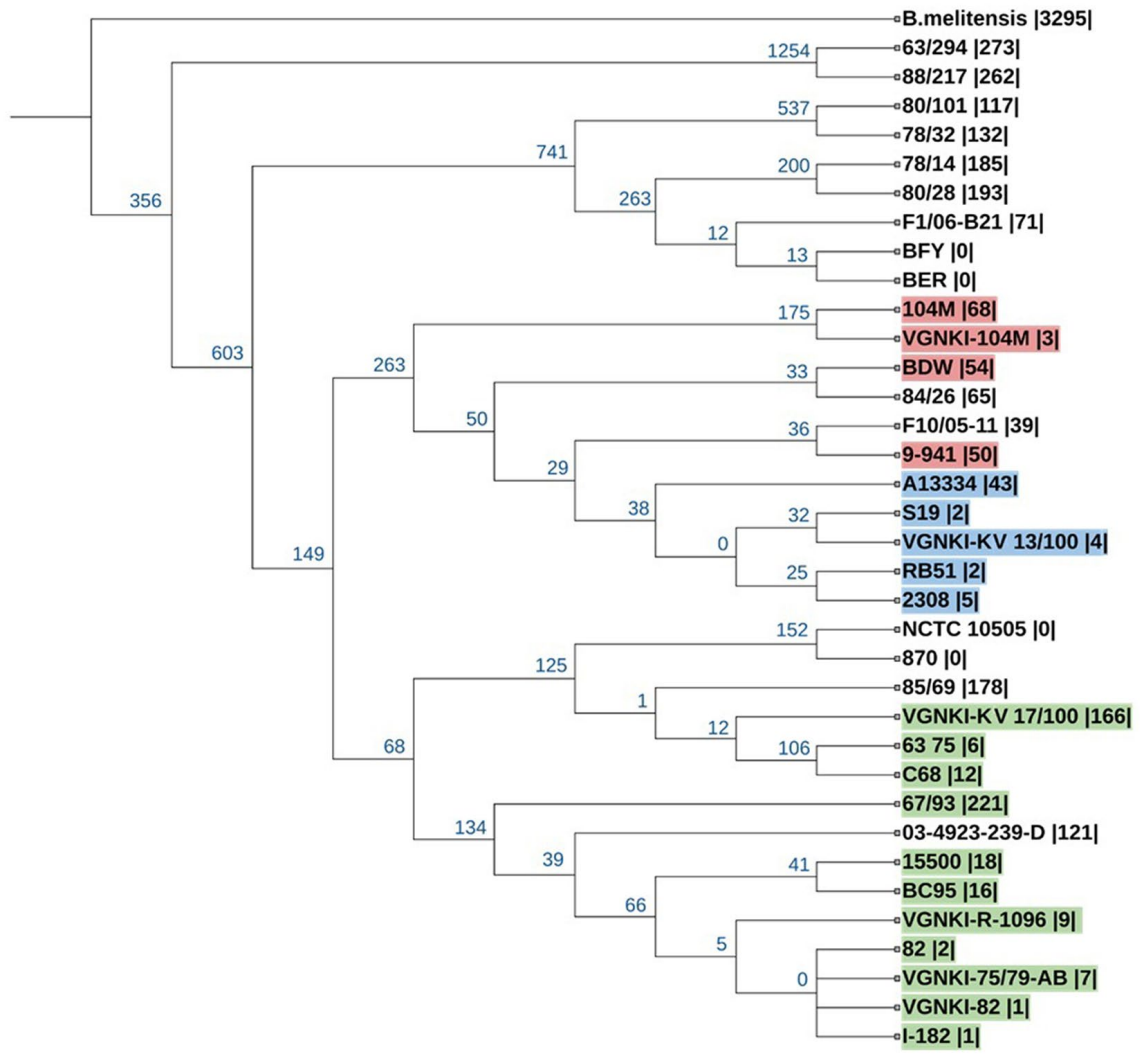
Phylogenetic tree of *Brucella abortus* for 11,510 SNPs. The prefix “VGNKI” indicates strains sequenced in this study. Numbers in blue at the roots of nodes show the number of SNPs that are shared exclusively among the descendants of each node. The number of unique SNPs for the genome is given in parentheses. Strains of ST-1 are highlighted in red, ST-2—in green, and ST-5—in blue.

To construct a phylogenetic tree based on SNP loci, we selected the genomes of vaccine strains and field isolates from the NCBI database that were assembled completely or at the scaffold level and added 6 vaccine strains from the All-Russian State Collection of Microorganisms Used in Veterinary Medicine and Animal Husbandry. The list of strains from the NCBI database is presented in Table 1. Pan-genome analysis was carried out using the program kSNP v.3.1. In total, we identified 14,768 SNPs, of which 11,510 are core SNPs. The rooted tree of all SNPs was constructed using the maximum parsimony method (Figure. 3). When constructing the tree, the genome of *Brucella melitensis bv*. 1 str. 16 M (NC_003317.1) was used as an outgroup.

Vaccine strain KV 13/100 (ST-5) belong to the same clade as vaccine strain S19 and it is a close relative to the vaccine strain RB-51. Vaccine strain 104 M (ST-1) is more distant relative to afore mentioned vaccine strains. Vaccine strains R-1096, 75/79AB and 82 (ST-2) belong to the same clade and very similar to each other. They are related distantly to vaccine strain KV 17/100-VGNKI (ST-2). It should be noted that the vaccine strain 104 M transferred from Soviet Russia to China 60 years ago (60) has acquired more than 60 unique SNPs compared to the Russian collection strain.

### Analysis of mutations in the virulence genes of industrial *Brucella abortus* strains

The complete genome sequence of *B. abortus* provides an important resource for future studies of pathogenicity determinants and virulence phenotypes of these bacteria, as well as serves as a basis for quality control of medicinal products for animals (29).

We analyzed the nucleotide sequence of virulence genes in our vaccine strains using the list of *Brucella* virulence genes from VFDB and the Victors database. For every SNP, we estimated its possible contribution to protein function using SIFT (61), then additionally checked the protein conservation level in the mutation site using UniProt (62). We found alterations and mutations that could explain the attenuated virulence in all six vaccine strains (Table 6).

**Table 6:** The mutations observed in virulence genes of vaccine strains.

Virulence genes	R-1096	75/79-AB	82	KV 17/100	KV 13/100	104 M
*pmm*	–	–	–	N255K	Partial deletion	–
*wbdA*	Deletion	–	–	–	–	–
*wbkA*	–	–	–	**G225V**, A321E	–	–
*wboA*	Deletion	**W219L**	**W219L**	–	–	–
*eryB*	–	–	–	–	–	W215X

The bold font indicates missense mutations occurred in conservative sites of proteins.

So, the virulence genes *wbdA* and *wboA* is absent in the vaccine strain R-1096. The IS711-like insertion sequence destroys the *pmm* gene in the vaccine strain 13/100. The *eryB* gene in strain 104 M codes a non-functional truncated protein because of nonsense mutation W215X. The attenuated phenotype of the strains KV 17/100, 75/79-AB, and 82 can be explained by missense mutations that occur in conservative sites of functional domains in virulence proteins. The strain KV 17/100 has a substitution mutation G255V in a very conservative site of the glycosyl transferase domain of the wbkA protein (Supplementary Figure S1). Both strains 75/79-AB and 82 have a substitution W219L located in a conservative site of the glycosyl transferase domain of the *wboA* protein (Supplementary Figure S2). The strain KB 17/100 has two substitutions N255K in the *pmm* gene and A321 in the *wbkA* gene, which can also negatively impact the function of corresponding proteins because they are in semi-conservative sites (Supplementary Figures S3, S4).

Thus, the five vaccine strains analyzed here are deficient in classical virulence genes responsible for the biosynthesis of LPS O-chain: *pmm*, *wbdA*, *wbkA*, *wboA* (63). These mutations can be responsible for these vaccine strains’ R-form type of colonies (Table 3). The deficiency of the *wboA* gene observed here in strains R-1096, 75/79-AB, and 82 was reported earlier for vaccine strain RB51 (64).

Yu et al. (22) described a set of candidate genes associated with virulence attenuation in vaccine strain 104 M. We complement this set with gene *eryB*, whose function is impaired by preliminary stop codon W215X. The gene *eryB* is a part of operon eryABCD encoding the erythritol dissimilative pathway (65, 66) reported that the growth of vaccine strain B19 was inhibited by erythritol due to mutations in genes *eryC* and e*ryD*.

## Conclusion

This study represents a comprehensive bioinformatics analysis of genomes of 6 *Brucella* vaccine strains. The results of our study serve as a prerequisite for improving the diagnostics of brucellosis in animals and will allow to reduce the risks associated with the spread of this infection in Russia, as well as to improve the control over the quality and safety of anti-brucellosis immunobiological agents.

## Data availability statement

The datasets presented in this study can be found in online repositories. The name of the repository and accession number can be found below: NCBI; PRJNA932701, https://www.ncbi.nlm.nih.gov/.

## Ethics statement

The study was approved by the Ethics Committee of VGNKI.

## Author contributions

AP: project management and securing funding. OS and AM: conceptualization and methodology. IS and IT: molecular genetic studies. AB and VG: bioinformatics analysis. OP, EK, and OS: writing and editing. All authors contributed to the article and approved the submitted version.

## Funding

This work was supported by the grant # 075-15-2021-1053 from the Ministry of Science and Higher Education of the Russian Federation.

## Conflict of interest

The authors declare that the research was conducted in the absence of any commercial or financial relationships that could be construed as a potential conflict of interest.

## Publisher’s note

All claims expressed in this article are solely those of the authors and do not necessarily represent those of their affiliated organizations, or those of the publisher, the editors and the reviewers. Any product that may be evaluated in this article, or claim that may be made by its manufacturer, is not guaranteed or endorsed by the publisher.

## References

[1] Bang B. Stribold. The etiology of contagious abortion Zeitschr.f. Tiermed, (1897). 1:103–105.

[2] Corner LA Alton GG. Persistence of *Brucella abortus* strain 19 infection in adult cattle vaccinated with reduced doses. Res Vet Sci. (1981) 31:342–4. doi: 10.1016/S0034-5288(18)32468-8, PMID: 6805054

[3] Smith R Smoll F Schutz R. Measurement and correlates of sport-specific cognitive and somatic trait anxiety: the sport anxiety scale. Anxiety Res. (1990) 2:263–80. doi: 10.1080/08917779008248733

[4] DelVecchio VG Kapatral V Redkar Guy Patra RJ Mujer C Los T Ivanova N. The genome sequence of the facultative intracellular pathogen Brucella melitensis. Proc Natl Acad Sci. (2002) 99:443–8. doi: 10.1073/pnas.22157539 11756688 PMC117579

[5] Richard C Essenberg RS Nelson K Paulsen I. Sugar metabolism by Brucellae. Vet Microbiol. (2002) 90:249–61. doi: 10.1016/S0378-1135(02)00212-2 12414147

[6] Gopalakrishnan A Dimri U Saminathan M Yatoo MY Priya BG Gopinath D. Virulence factors, intracellular survivability and mechanism of evasion from host immune response by *Brucella*: an overview. J Anim Plant Sci. (2016) 26:1542–55.

[7] Lapaque N Moriyon I Moreno E Gorvel J-P. *Brucella* lipopolysaccharide acts as a virulence factor. Curr Opin Microbiol. (2005) 8:60–6. doi: 10.1016/j.mib.2004.12.003, PMID: 15694858

[8] Martirosyan A Moreno E Gorvel J. An evolutionary strategy for a stealthy intracellular *Brucella* pathogen. Immunol Rev. (2011) 240:211–34. doi: 10.1111/j.1600-065X.2010.00982.x, PMID: 21349096

[9] Martirosyan A Pérez-Gutierrez C Banchereau R Dutartre H Lecine P Dullaers M. *Brucella* β 1,2 cyclic glucan is an activator of human and mouse dendritic cells. PLoS Pathog. (2012) 8:e1002983. doi: 10.1371/journal.ppat.1002983, PMID: 23166489 PMC3499565

[10] Martin R. Regional economic resilience, hysteresis and recessionary shocks. J Econ Geogr. (2012) 12:1–32. doi: 10.1093/jeg/lbr019

[11] Lim JJ Kim DH Lee JJ Kim DG Min W Lee HJ. Evaluation of recombinant 28 kDa outer membrane protein of *Brucella abortus* for the clinical diagnosis of bovine brucellosis in Korea. J Vet Med Sci. (2012) 74:687–91. doi: 10.1292/jvms.11-0512 22214857

[12] Posadas DM Ruiz-Ranwez V Bonomi HR Martín FA Zorreguieta A. BmaC, a novel autotransporter of *Brucella suis*, is involved in bacterial adhesion to host cells. Cell Microbiol. (2012) 14:965–82. doi: 10.1111/j.1462-5822.2012.01771.x 22321605

[13] Del Giudice MG Ugalde JE Czibener C. A lysozyme-like protein in *Brucella abortus* is involved in the early stages of intracellular replication. Infect Immun. (2013) 81:956–64. doi: 10.1128/IAI.01158-12 23319555 PMC3584897

[14] Ruiz-Ranwez V Posadas DM Estein SM Abdian PL Martin FA Zorreguieta A. The BtaF trimeric autotransporter of *Brucella suis* is involved in attachment to various surfaces, resistance to serum and virulence. PLoS One. (2013) 8:e79770. doi: 10.1371/journal.pone.0079770, PMID: 24236157 PMC3827427

[15] Lee JJ Kim JH Kim DG Kim DH Simborio HL Min WG. Characterization of betaine aldehyde dehydrogenase (BetB) as an essential virulence factor of *Brucella abortus*. Vet Microbiol (2014); 168, 131–140. doi: 10.1016/j.vetmic.2013.10.007 24210811

[16] Mirabella A Terwagne M Zygmunt MS Cloeckaert A De Bolle X Letesson JJ. *Brucella melitensis* MucR, an orthologue of *Sinorhizobium meliloti* MucR, is involved in resistance to oxidative, detergent, and saline stresses and cell envelope modifications. J Bacteriol. (2013) 195:453–65. doi: 10.1128/JB.01336-12, PMID: 23161025 PMC3554010

[17] Mancilla M. Smooth to rough dissociation in *Brucella*: the missing link to virulence/*M. Mancilla*. Front Cell Infect Microbiol. (2016) 5:98. doi: 10.3389/fcimb.2015.00098 26779449 PMC4700419

[18] Scholz HC Hubalek Z Sedlácek I Vergnaud G Tomaso H Al Dahouk S. *Brucella microti* sp. nov., isolated from the common vole *Microtus arvalis*. Int J Syst Evol Microbiol. (2008) 58:375–82. doi: 10.1099/ijs.0.65356-0, PMID: 18218934

[19] Foster G Osterman BS Godfroid J Jacques I Cloeckaert A. *Brucella ceti* sp. nov. and *Brucella pinnipedialis* sp. nov. for *Brucella* strains with cetaceans and seals as their preferred hosts. Int J Syst Evol Microbiol. (2007) 57:2688–93. doi: 10.1099/ijs.0.65269-0, PMID: 17978241

[20] Scholz HC Revilla-Fernández S Dahouk SA Hammerl JA Zygmunt MS Cloeckaert A. *Brucella vulpis* sp. nov., isolated from mandibular lymph nodes of red foxes (*Vulpes vulpes*). Int J Syst Evol Microbiol. (2016) 66:2090–8. doi: 10.1099/ijsem.0.000998, PMID: 26928956

[21] Verger JM Grimont F Grimont PA Grayon M. Taxonomy of the genus *Brucella*. Ann Inst Pasteur Microbiol. (1987) 138:235–8. doi: 10.1016/0769-2609(87)90199-2 3606880

[22] Yu D Hui Y Zai X Xu J Liang L Wang B. Comparative genomic analysis of *Brucella abortus* vaccine strain 104M reveals a set of candidate genes associated with its virulence attenuation. Virulence. (2015) 6:745–54. doi: 10.1080/21505594.2015.1038015, PMID: 26039674 PMC4826108

[23] Halling SM Peterson-Burch BD Bricker BJ Zuerner RL Qing Z Li LL. Completion of the genome sequence of *Brucella abortus* and comparison to the highly similar genomes of *Brucella melitensis* and *Brucella suis*. J Bacteriol. (2005) 187:2715–26. doi: 10.1128/JB.187.8.2715-2726.2005, PMID: 15805518 PMC1070361

[24] Minogue TD Daligault HA Davenport KW Bishop-Lilly KA Broomall SM Bruce DC. Whole-genome sequences of 24 *Brucella* strains. Genome Announc. (2014) 2:e00915–4. doi: 10.1128/genomeA.00915-14 25237024 PMC4172273

[25] Paradiso R Orsini M Criscuolo D Borrelli R Valvini O Cammà C. Complete genome sequencing of 10 *Brucella abortus* Biovar 3 strains isolated from water Buffalo. Genome Announc. (2018) 6:e00180–18. doi: 10.1128/genomeA.00180-18 29674531 PMC5908924

[26] Shevtsov A Tarlykov P Zholdybayeva E Shevtsova E Momynkulov D Sytnik I. Draft genome sequence of the live vaccine strain *Brucella abortus* 82. Genome Announc. (2013) 1:e01101–13. doi: 10.1128/genomeA.01101-13 24371203 PMC3873613

[27] Bricker B Goonesekere N Bayles D Alt D Olsen S Vrentas C. Genome report—a genome sequence analysis of the RB51 strain of *Brucella abortus* in the context of its vaccine properties. G3: genes, genomes. Genetics. (2020) 10:1175–81. doi: 10.1534/g3.119.400964 PMC7144086 32111651

[28] Kim H Jeong W Jeoung HY Song JY Kim JS Beak JH. Complete genome sequence of *Brucella abortus* A13334, a new strain isolated from the fetal gastric fluid of dairy cattle. J Bacteriol. (2012) 194:5444. doi: 10.1128/JB.01124-12, PMID: 22965076 PMC3457244

[29] Crasta OR Folkerts O Fei Z Mane SP Evans C Martino-Catt S. Genome sequence of *Brucella abortus* vaccine strain S19 compared to virulent strains yields candidate virulence genes. PLoS One. (2008) 3:e2193. doi: 10.1371/journal.pone.0002193, PMID: 18478107 PMC2364660

[30] Chain PS Comerci DJ Tolmasky ME Larimer FW Malfatti SA Vergez LM. Whole-genome analyses of speciation events in pathogenic Brucellae. Infect Immun. (2005) 73:8353–61. doi: 10.1128/IAI.73.12.8353-8361.2005, PMID: 16299333 PMC1307078

[31] Andrews S (2010). FastQC: a quality control tool for high throughput sequence data. Available at: http://www.bioinformatics.babraham.ac.uk/projects/fastqc/

[32] Bolger AM Lohse M Usadel B. Trimmomatic: a flexible trimmer for Illumina sequence data. Bioinformatics. (2014) 30:2114–20. doi: 10.1093/bioinformatics/btu170, PMID: 24695404 PMC4103590

[33] Bankevich A Nurk S Antipov D Gurevich AA Dvorkin M Kulikov AS. SPAdes: a new genome assembly algorithm and its applications to single-cell sequencing. J Comput Biol. (2012) 19:455–77. doi: 10.1089/cmb.2012.0021, PMID: 22506599 PMC3342519

[34] Gurevich A Saveliev V Vyahhi N Tesler G. QUAST: quality assessment tool for genome assemblies. Bioinformatics. (2013) 29:1072–5. doi: 10.1093/bioinformatics/btt086, PMID: 23422339 PMC3624806

[35] Darling AC Mau B Blattner FR Perna NT. Mauve: multiple alignment of conserved genomic sequence with rearrangements. Genome Res. (2004) 14:1394–403. doi: 10.1101/gr.2289704, PMID: 15231754 PMC442156

[36] Hasman H Saputra D Sicheritz-Ponten T Lund O Svendsen CA Frimodt-Møller N. Rapid whole-genome sequencing for detection and characterization of microorganisms directly from clinical samples. J Clin Microbiol. (2014) 52:139–46. doi: 10.1128/JCM.02452-13 24172157 PMC3911411

[37] Larsen MV Cosentino S Rasmussen S Friis C Hasman H Marvig RL. Multilocus sequence typing of total-genome-sequenced bacteria. J Clin Microbiol. (2012) 50:1355–61. doi: 10.1128/JCM.06094-11 22238442 PMC3318499

[38] Brettin T Davis JJ Disz T Edwards RA Gerdes S Olsen GJ. RASTtk: a modular and extensible implementation of the RAST algorithm for building custom annotation pipelines and annotating batches of genomes. Sci Rep. (2015) 10:8365. doi: 10.1038/srep08365 PMC4322359 25666585

[39] Zankari E Hasman H Cosentino S Vestergaard M Rasmussen S Lund O. Identification of acquired antimicrobial resistance genes. J Antimicrob Chemother. (2012) 67:2640–4. doi: 10.1093/jac/dks261, PMID: 22782487 PMC3468078

[40] Gupta SK Padmanabhan BR Diene SM Lopez-Rojas R Kempf M Landraud L. ARG-ANNOT, a new bioinformatic tool to discover antibiotic resistance genes in bacterial genomes. Antimicrob Agents Chemother. (2014) 58:212–20. doi: 10.1128/AAC.01310-13 24145532 PMC3910750

[41] Available at: https://github.com/tseemann/abricate (дата обращения 20 11 2022).

[42] Liu B Zheng D Jin Q Chen L Yang J. VFDB 2019: a comparative pathogenomic platform with an interactive web interface. Nucleic Acids Res. (2019) 47:D687–92. doi: 10.1093/nar/gky1080, PMID: 30395255 PMC6324032

[43] Sayers S Li L Ong E Deng S Fu G Lin Y. Victors: a web-based knowledge base of virulence factors in human and animal pathogens. Nucleic Acids Res. (2019) 47:D693–700. doi: 10.1093/nar/gky999, PMID: 30365026 PMC6324020

[44] Cury J Jové T Touchon M Néron B Rocha EP. Identification and analysis of integrons and cassette arrays in bacterial genomes. Nucleic Acids Res. (2016) 44:4539–50. doi: 10.1093/nar/gkw319, PMID: 27130947 PMC4889954

[45] Gardner SN Slezak T Hall BG. kSNP3. 0: SNP detection and phylogenetic analysis of genomes without genome alignment or reference genome. Bioinformatics. (2015) 31:2877–8. doi: 10.1093/bioinformatics/btv271 25913206

[46] Letunic I Bork P. Interactive tree of life (iTOL) v5: an online tool for phylogenetic tree display and annotation. Nucleic Acids Res. (2021) 49:W293–6. doi: 10.1093/nar/gkab301 33885785 PMC8265157

[47] Buck JM. Studies of vaccination during calfhood to prevent bovine infectious abortion. J Agric Res. (1930) 41:667–89.

[48] Elberg SS Steiner PE Doll JP. Immunization against *Brucella* infection. V. Histopathologic appraisal of immunity induced in mice by a streptomycin-dependent mutant of *Brucella melitensis*. Am J Pathol. (1955) 31:1065–75. 13268611 PMC1942601

[49] Schurig GG Roop RM II Bagchi T Boyle S Buhrman D Sriranganathan N. Biological properties of RB51; a stable rough strain of *Brucella abortus*. Vet Microbiol. (1991) 28:171–88. doi: 10.1016/0378-1135(91)90091-S, PMID: 1908158

[50] Shumilov KV. Biological properties of the vaccine strain *B. abortus* 104 M. Proc VIEV. (1983) 57:42–7.

[51] Salmakov KM. Live vaccine against brucellosis from strain 82. Vet Med. (1975) 43–5. 1216595

[52] RU2113857 C1 Nikiforov I.P. Shumilov K. V. Kalmykov V.V. Klimanov A.I. Shumilov K. V. Shumilov K. V.. Vaccine against brucellosis in cattle. (1997).

[53] RU 2130068 C1 Kalmykov V.V. Shumilov K. V.. (1999). *Brucella abortus* strain used for the manufacture of diagnostic and preventive biological preparations against animal brucellosis, Antrag Nr. 97118640/13 vom 12. Nov. 1997.

[54] RU 2149184 C1 Kalmykov V.V.. (2000). A method is known for evaluating the immunogenicity of anti-brucellosis vaccine strains by determining the rate of elimination from the body of immunized animals of the *Brucella* control strain *B. abortus* KB 13/100-DEPT.

[55] SU 946039 А Salmakov К.М. (1979). A method for preparing an antigen for the differential serological diagnosis of brucellosis in animals, including obtaining a bacterial mass of the *B. abortus* R-1096 strain and inactivating it by gamma irradiation.

[56] Khurana SK Sehrawat A Tiwari R Prasad M Gulati B Shabbir MZ. Bovine brucellosis—a comprehensive review. Vet Q. (2021) 41:61–88. doi: 10.1080/01652176.2020.1868616, PMID: 33353489 PMC7833053

[57] https://www.центр-ветеринарии.рф.

[58] Shumilov KV Sklyarov O Klimanov A. Designing vaccines against cattle brucellosis. Vaccine. (2010) 28:F31–4, ISSN 0264-410X, doi: 10.1016/j.vaccine.2010.03.049, PMID: 20362619

[59] Seleem M Boyle S Sriranganathan N. Brucellosis: a re-emerging zoonosis. Vet Microbiol. (2009) 140:392–8. doi: 10.1016/j.vetmic.2009.06.021 19604656

[60] Li ZQ Shi JX Fu WD Zhang Y Zhang J Wang Z. A *Brucella melitensis* M5-90 wboA deletion strain is attenuated and enhances vaccine efficacy. Mol Immunol. (2015) 66:276–83. doi: 10.1016/j.molimm.2015.04.004, PMID: 25899866

[61] Ng PC Henikoff S. SIFT: predicting amino acid changes that affect protein function. Nucleic Acids Res. (2003) 31:3812–4. doi: 10.1093/nar/gkg509, PMID: 12824425 PMC168916

[62] UniProt Consortium. UniProt: a hub for protein information. Nucleic Acids Res. (2015) 43:D204–12. doi: 10.1093/nar/gku989, PMID: 25348405 PMC4384041

[63] Delrue RM Lestrate P Tibor A Letesson JJ De Bolle X. *Brucella* pathogenesis, genes identified from random large-scale screens. FEMS Microbiol Lett. (2004) 231:1–12. doi: 10.1016/S0378-1097(03)00963-7 14979322

[64] Vemulapalli R McQuiston JR Schurig GG Sriranganathan N Halling SM Boyle SM. Identification of an IS 711 element interrupting the wboA gene of *Brucella abortus* vaccine strain RB51 and a PCR assay to distinguish strain RB51 from other *Brucella* species and strains. Clin Diagn Lab Immunol. (1999) 6:760–4. doi: 10.1128/CDLI.6.5.760-764.1999, PMID: 10473532 PMC95769

[65] Sangari FJ Agüero J García-Lobo JM. The genes for erythritol catabolism are organized as an inducible operon in *Brucella abortus*. Microbiology. (2000) 146:487–95. doi: 10.1099/00221287-146-2-487 10708387

[66] Sangari FJ García-Lobo JM Agüero J. The *Brucella abortus* vaccine strain B19 carries a deletion in the erythritol catabolic genes. FEMS Microbiol Lett. (1994) 121:337–42. doi: 10.1111/j.1574-6968.1994.tb07123.x, PMID: 7926690

