# Supplementary material for: Russian collection of Brucella abortus vaccine strains: annotation, implementation and genomic analysis
Source: Front Vet Sci. 2023 Jun 21;10:1154520. doi: 10.3389/fvets.2023.1154520 (PMC10322197; doi:10.3389/fvets.2023.1154520)
Supplement: Supplementary file 1 [file Image_1.pdf]

Mutations in conservative sites

wbkA, G225V

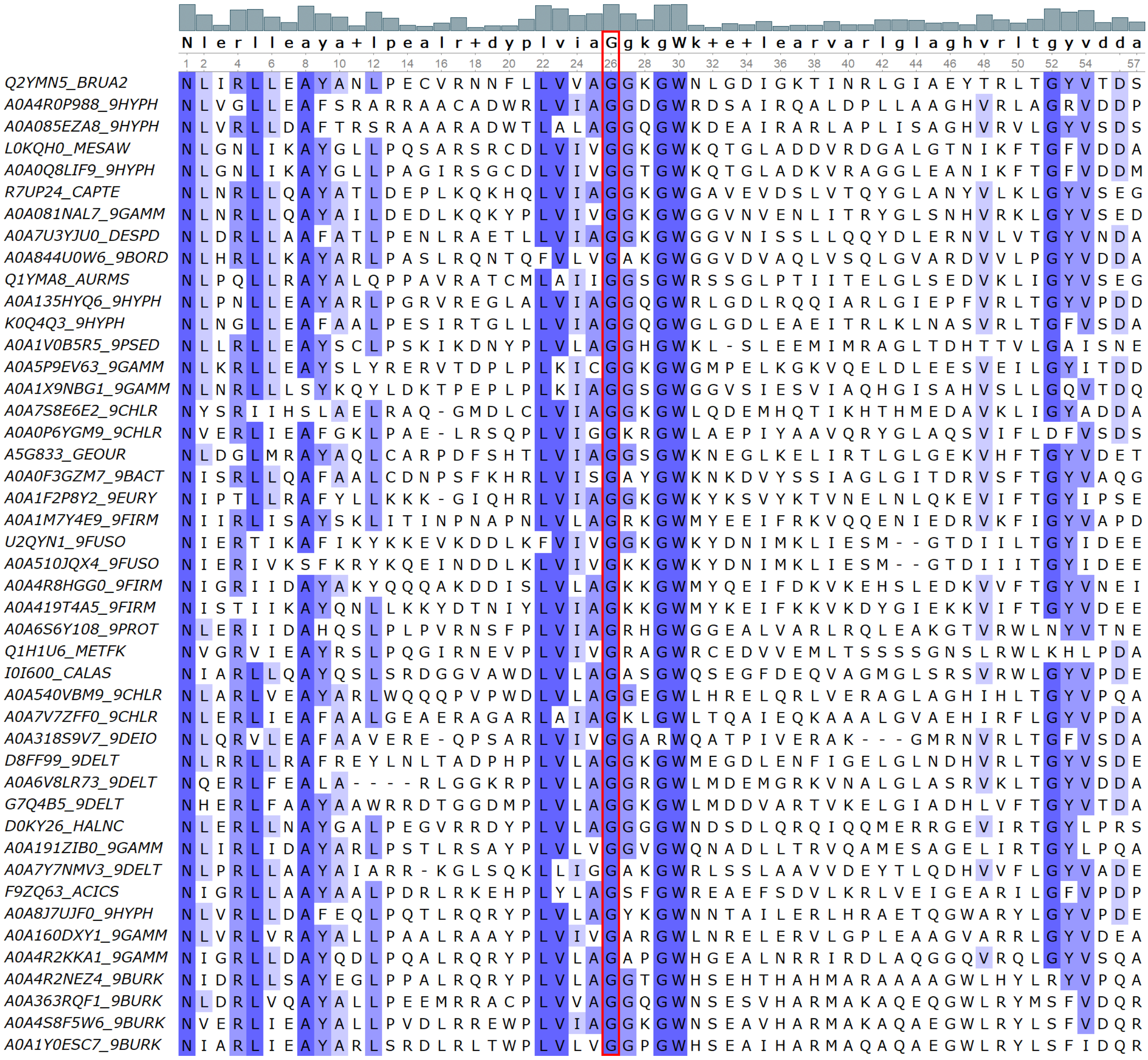

Supplemental Figure 1. The multiple alignment of bacterial glycosyltransferases from UniProt database which are homologous to the wbkA protein of Brucella abortus (Q2YMN5_BRUA2). The site of substitution G255V observed in KB 17/100 strain is indicated by red rectangle. The intensity of blue color corresponds to conservation level.

wboA, W219L

Glycosyl transferase domain (InterPro entry IPR001296)

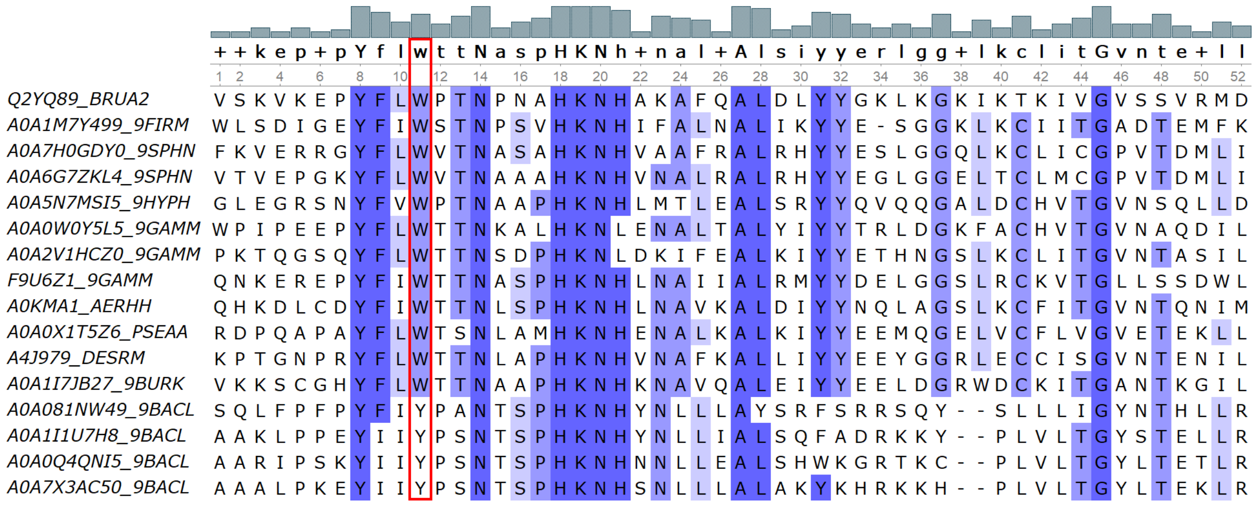

Supplemental Figure 2. The multiple alignment of bacterial glycosyltransferases from UniProt database which are homologous to the wboA protein of Brucella abortus (Q2YQ89_BRUA2). The site of substitution W219L observed in strains 75/79-AB and 82 is indicated by red rectangle. The intensity of blue color corresponds to conservation level.

Mutations in semi-conservative sites

Pmm, N255K

Alpha-D-phosphohexomutase, alpha/beta/alpha domain III (InterPro entry IPR005846)

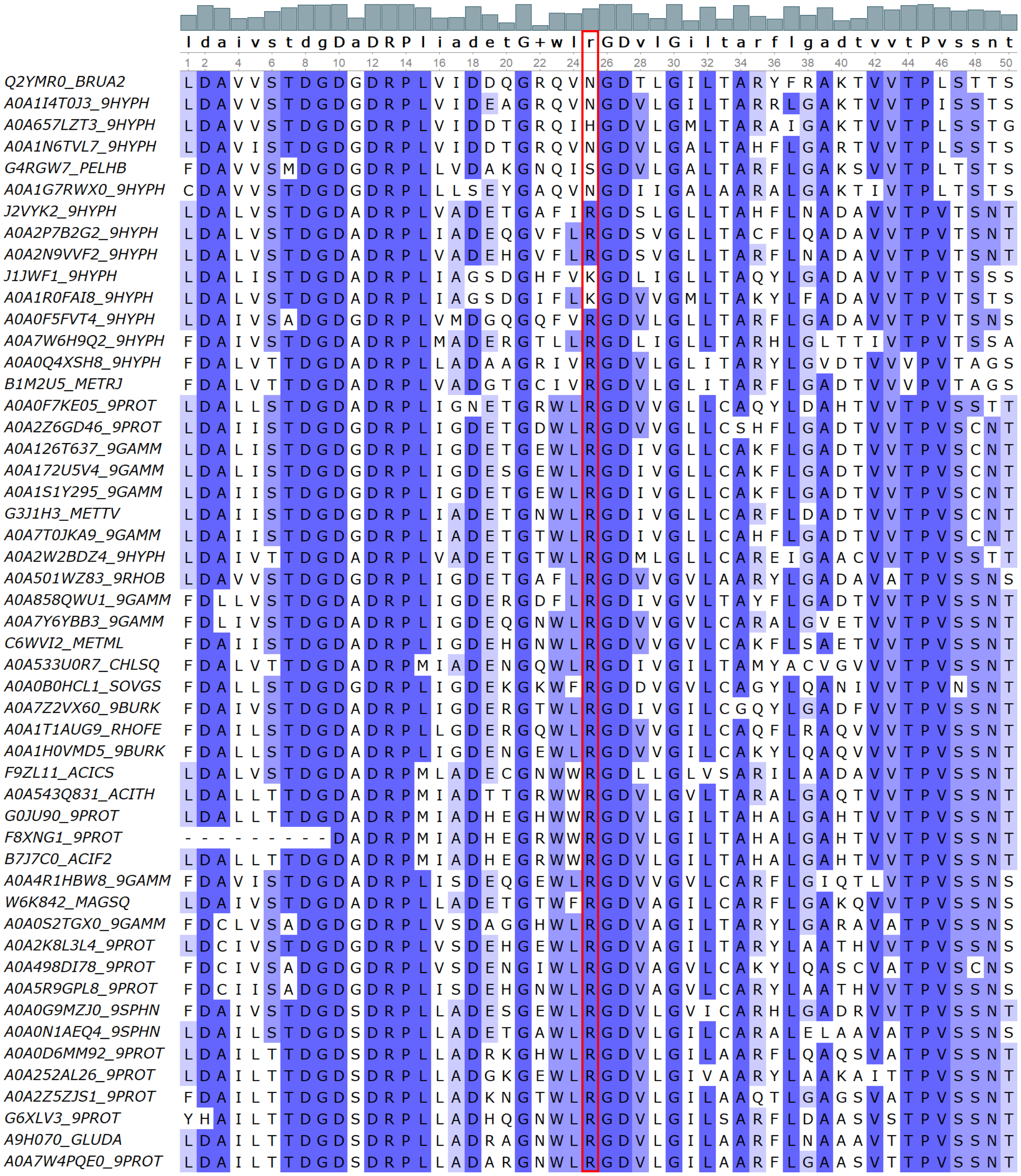

Supplemental Figure 3. The multiple alignment of bacterial phosphomannomutases from UniProt database which are homologous to the pmm protein of Brucella abortus (Q2YMR0_BRUA2). The site of substitution N255K observed in strain KB 17/100 is indicated by red rectangle. The intensity of blue color corresponds to conservation level.

wbkA, A321E

Glycosyl transferase family 1 domain (IPR001296)

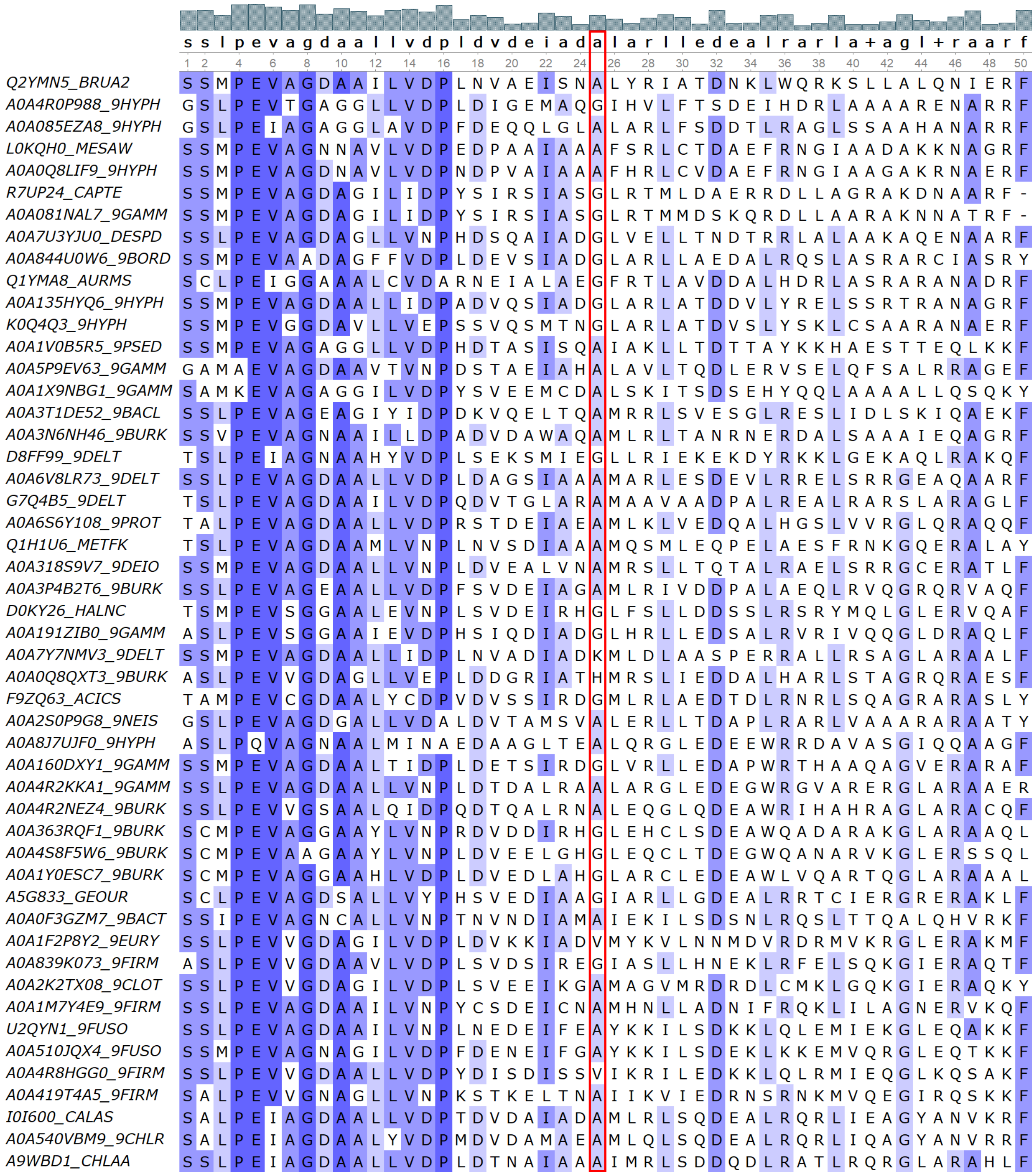

Supplemental Figure 4. The multiple alignment of bacterial glycosyltransferases from UniProt database which are homologous to the wbkA protein of Brucella abortus (Q2YMN5_BRUA2). The site of substitution A321E observed in strain KB 17/100 is indicated by red rectangle. The intensity of blue color corresponds to conservation level.
